# The effect of weather variables on the severity, duration, and frequency of headache attacks in the cases of episodic migraine and episodic tension-type headache

**DOI:** 10.3906/sag-2004-66

**Published:** 2021-06-28

**Authors:** Nergis AKGÜN, Esra ACIMAN DEMİREL, Mustafa AÇIKGÖZ, Ulufer ÇELEBİ, Fürüzan KÖKTÜRK, Hüseyin Tuğrul ATASOY2

**Affiliations:** 1 Department of Neurology, Zonguldak Atatürk State Hospital, Zonguldak Turkey; 2 Department of Neurology, Faculty of Medicine, Zonguldak Bülent Ecevit University, Zonguldak Turkey; 3 Department of Biostatistics, Faculty of Medicine, Zonguldak Bülent Ecevit University, Zonguldak Turkey

**Keywords:** Headache, migraine, tension-type headache, weather

## Abstract

**Background/aim:**

Although many headache patients report that the frequency and severity of attacks vary according to the season or weather in clinical practice, the relationship between the characteristics of the attacks and the weather is not very clear in episodic headaches. We aimed to compare the effects of weather variables (temperature, wind speed, wind direction, humidity, pressure, ultraviolet index, and sunshine duration) on episodic migraine (EM) and episodic tension-type headache (ETTH) attacks (incidence, duration, and severity).

**Materials and methods:**

Fifty patients with EM and fifty patients with ETTH diagnosed according to International Classification of Headache Disorders-II are included in the study. Patients were given one diary for headache follow-up. The evaluation form on the relationship between the duration, frequency, and severity of the pain and the findings obtained from the headache diaries were compared with the daily weather data, and the two headache groups were compared with each other in terms of the effect of meteorological data on the pain characteristics.

**Results:**

It is determined that mean wind velocity in EM attacks is significantly higher when compared to the tension-type headache (TTH) attacks and mean UV index is significantly higher in TTH attacks (p = 0.018 and 0.039). Mean UV index in TTH attack days was reported higher in women than men (p = 0.044). Mean sunshine duration in TTH attack days was reported longer in women than men (p = 0.050). When mean age gets higher in patients with migraine, mean temperature in the days of attack gets lower (r = –0.146 and p = 0.046).

**Conclusion:**

During the treatment of migraine and TTH patients, recommendations and warnings about weather conditions can be made. This information can guide patients to regulate their daily living activities. The importance of considering the weather-headache relationship during the review of the current treatment in cases of unresponsiveness to treatment should be kept in mind.

## 1. Introduction

Headache is one of the most common medical complaints with a lifetime prevalence of 95% in women and 90% in men [1]. Migraine and tension-type headache (TTH) are the two most common forms of headache [2]. According to World Health Organization (WHO), migraine is one of the diseases that causes the most limitations [3]. TTH is the most common primary headache type, and its lifetime prevalence in the general population varies between 30% and 78% in various studies [4].

Studies have often investigated the relationship between migraine patients and weather conditions [5,6,7,8,9,10,11]. However, studies on episodic headache types are rather limited to our knowledge [12,13]. A retrospective study, conducted by Yilmaz et al. with 3491 patients who admitted to the emergency department with the diagnosis of migraine, found that the number of migraine patients admitted to the emergency department increased in case of high temperatures and low humidity [5]. In one study, it was stated that weather changes triggered or increased the severity of headache in migraine patients [6]. Some studies showed that various air parameters including ambient temperature, barometric pressure, relative humidity, and wind speed may be associated with headache [8,9,14,15,16]. In many studies, no statistically significant relationship was found between migraine headaches and weather parameters [17,18,19,20,21].

In the present study, we tried to determine the effects of weather variables (temperature, wind speed, wind direction, humidity, pressure, ultraviolet (UV) index and sunshine duration) on the characteristics of episodic migraine and episodic tension-type headache (ETTH) attacks (frequency, duration, and severity). We aimed to examine whether the effects of weather variables differ between these two types of episodic headache attacks and their relationship with sex and age.

## 2. Materials and methods

### 2.1. Study design and participants

Our research has started in compliance with the Helsinki Declaration resolutions, patient rights regulation and codes of conduct after the approval given with the meeting resolution dated 17.06.2014 and numbered 2014/12 taken in Bülent Ecevit University Application and Research Hospital which was the local ethics committee.

This prospective study included 50 episodic migraine and 50 episodic tension type headache patients who accepted to participate in the study, who were diagnosed according to International Classification of Headache Disorders (ICHD-3), were resident in Zonguldak province, were at least literate and over 18 years of age, and were admitted to neurology outpatient clinic due to headache between the dates 20.07.2014 and 20.04.2015. Patients with any headaches other than episodic migraine and episodic tension type headache and patients with incomplete data were excluded from the study. 

Written approval form was taken from patients who agreed to participate in the study. Neurological evaluations of the subjects were performed and they were made to complete the Headache Assessment Form used in our clinic. Headache questionnaire was performed by using the Headache Assessment Form used in our clinic. The patients were given a diary. At the end of our study, through the evaluation of these diaries we analysed the relationship between the duration, frequency, and severity of pain and the obtained daily weather data. 50 episodic migraine patients and 50 episodic TTH patients were compared in terms of their descriptive characteristics. One hundred eighty-eight headache attack recorded by 50 episodic migraine patients and 233 headache attack recorded by 50 TTH patients were taken into account when two group was compared in terms of the relationship between attack duration, severity, and weather condition parameters. In addition, with the analysis of 188 migraine attack, the relationship between weather condition and age, and the relationship between weather condition and sex, and with the analysis of 233 TTH attack, the relationship between weather condition and age and the relationship between weather condition and sex were examined. In our study, the effect of weather parameters on the duration and severity of 188 migraine attack and the duration and severity of 233 TTH attack were also analysed.

During the nine-month period of our study, meteorological parameters were obtained from the data of Republic of Turkey Ministry of Agriculture and Forestry showing the weather conditions of Zonguldak (http://www.dmi.gov.tr/tahmin/il-ve-ilceler.aspx?m=zonguldak#sfb) at 12.00 noon every day and via the recordings of two separate international weather forecast organizations which are Weatheronline (http://www.weatheronline.co.uk/turkey/zonguldak.htm) and Meteocentrale (http://www.meteocentrale.ch/index.php?l=0&id=1199&searchstring=zonguldak). Weather variables to be considered in the study were temperature, wind speed, wind direction, humidity, pressure, ultraviolet index, and sunshine duration.

The evaluation of the relationship between headache attacks and meteorological parameters of the patients included in the study was made through headache diaries kept by the patients.

### 2.2. Statistical analysis

Statistical analysis was performed using SPSS 19.0 program (IBM Corp., Armonk, NY, USA). The suitability of the continuous variables for normal distribution was examined by Shapiro–Wilk test. Descriptive statistics for continuous variables were expressed with mean ± standard deviation, and for categorical data, they were expressed with numbers and percentages. Differences between the groups in terms of categorical variables were examined by Chi-square test. When parametric test assumptions were provided for the comparison of the two groups in terms of continuous variables, the significance test of the difference between the two means was used, and when it was not provided, the Mann–Whitney U test was used. In comparison of three or more groups in terms of continuous variables, Kruskal–Wallis analysis of variance was used. The relationship between two continuous variables was analysed by Spearman correlation analysis and the p-value < 0.05 is considered significant.

## 3. Results

Descriptive characteristics of migraine and TTH patients included in the study are given in Table 1. All patients live in urban. 

**Table 1:** Descriptive characteristics of patients according to headache groups.

		Migrainen = 50	TTHn = 50	p
Age (years)		37.0 ± 11.3	34.3 ± 13.2	0.114
Number of children		2.4 ± 1.1	2.2 ± 1.0	0.568
Sex	Female	32 (64%)	40 (80%)	0.119
	Male	18 (36%)	10 (20%)	
Education	Primary school	6 (12%)	5 (10%)	0.147
	Middle School	7 (14%)	3 (6%)	
	High school	16 (32%)	10 (20%)	
	University	21 (42%)	32 (64%)	
Occupation	Student	5 (10%)	16 (32%)	0.033
	Civil Servant	17 (34%)	14 (28%)	
	Worker	10 (20%)	4 (8%)	
	Unemployed	18 (36%)	16 (32%)	
The effect of pain on work force	yes	31 (62%)	25 (50%)	0.314
	no	19 (38%)	25 (50%)	

TTH: Tension-type headache.*The values are presented as mean +/-SD or n (%).

Six of migraine patients had hypertension, two of them had diabetes mellitus, three of them had hyperlipidemia. One of TTH patients had hypertension. 

Four of migraine patients used beta blocker, one of them used selective serotonin reuptake inhibitors (SSRIs) and eight of them used tricyclic antidepressant for preventive medication. One of TTH patients used SSRIs for preventive medication.

The headache characteristics of the patients according to the groups are shown in Table 2.

**Table 2:** Headache characteristics of patients according to groups.

		Migrainen = 50	TTHn = 50	p
Time since headache onset (months)		103.7 ± 106.0	64.0 ± 73.9	0.014
VAS		7.0 ± 1.7	5.4 ± 2.3	0.001
Frequency	Everyday	8 (16%)	2 (4%)	0.023
	Few times a week	25 (50%)	19 (38%)	
	Few times a month	17 (34%)	29 (58%)	
Time	30 min-3 h	5 (10%)	33 (66%)	<0.001
	4-24 h	31 (62%)	16 (32%)	
	2 days or more	14 (28%)	1 (%2)	
Onset time	After getting up in the morning	21 (42%)	16 (32%)	0.040
	During the day	13 (26%)	25 (50%)	
	Night	16 (32%)	9 (18%)	
Onset Location	Right side of head	10 (20%)	13 (26%)	0.758
	Left side of head	11 (22%)	11 (22%)	
	Whole head	29 (58%)	26 (52%)	
Location	Right side of head	7 (14%)	8 (16%)	0.960
	Left side of head	8 (16%)	8 (16%)	
	Whole head	35 (70%)	34 (68%)	
Type of headache	Throbbing	22 (44%)	29 (58%)	0.230
	Feeling of head being pressed	28 (56%)	21 (42%)	
Nausea	Yes	29 (58%)	14 (28%)	0.005
	No	21 (42%)	36 (72%)	
Vomiting	Yes	12 (24%)	3 (6%)	0.025
	No	38 (76%)	47 (94%)	
Menstrual headache	Yes	7 (26.9%)	11 (28.2%)	1.000
	No	19 (73.1%)	28 (71.8%)	
The day of the menstrual headache	1st day	7 (87.5%)	9 (90%)	0.706
	2nd day	1 (12.5%)	0	
	3rd day	0	1 (10%)	

A total of 421 headache attacks were recorded by 50 migraine patients and 50 TTH patients in our study. In the comparison between the two groups in terms of headache duration, migraine attacks were found to last longer than TTH attacks and the difference between the two groups was found statistically significant (p < 0.001). In the comparison between the two groups in terms of headache severity, migraine attacks were found to be more severe than TTH attacks and the difference between the two groups was statistically significant (p < 0.001). However, the rate of the unuse of painkillers was 13.8% in migraine attacks and 46.4% in TTH attacks.

Headache attacks of groups were compared in terms of their relationship with air temperature, humidity, pressure, wind speed, wind direction, sunshine duration, and UV index. A significant difference was found between the two groups in terms of mean wind speed and mean UV index (p = 0.018 and p = 0.039, respectively). In migraine headaches, the mean wind speed on the attack days was significantly higher than the mean wind speed on the TTH attack days. The mean UV index was found to be significantly higher on TTH attack days.

The comparison of the headache attacks of the groups according to the weather parameters are shown in Table 3.

**Table 3:** Comparison of headache attacks according to weather parameters of groups

		Migrainen = 188 attacks	TTHn = 233 attacks	p
Temperature (°C)		10.3 ± 6.4	11.6 ± 7.1	0.059
Humidity percentage (%)		76.1 ± 20.0	75.6 ± 19.1	0.505
Pressure (mm Hg)		11.4 ± 1.0	1.0 ± 24.3	0.172
Wind speed (km/h)		7.4 ± 3.8	6.8 ± 4.2	0.018
Sunshine duration(h/y)		632.1 ± 95.9	650.1 ± 106.8	0.175
UV index		2.8 ± 1.5	3.2 ± 1.7	0.039
Winddirection	North	34 (18.1%)	34 (14.6%)	0.655
	South	26 (13.8%)	24 (10.3%)	
	East	30 (16%)	32 (13.7%)	
	West	33 (17.6%)	54 (23.2%)	
	Northwest	34 (18.1%)	46 (19.7%)	
	Northeast	14 (7.4%)	24 (10.3%)	
	Southeast	5 (2.7%)	6 (2.6%)	
	Southwest	12 (6.4%)	13 (5.6%)	

In the migraine and TTH attacks, the relationship between weather parameters and sex and age was investigated. As a result of the examination of the relationship between migraine attacks and weather conditions, no difference was found in terms of sex. However, the mean UV index in TTH attacks was found to be significantly higher in females than in males (p = 0.044). The mean sunshine duration in TTH attacks was also reported near significant longer in females than in males (p = 0.05) (Table 4). No significant difference was found between the sexes in the other parameters.

**Table 4:** The relationship between weather parameters and sex in TTH attacks.

	Female	Male	p
Temperature(°C)	11.7 ± 7.3	10.9 ± 6.2	0.536
Humidity percentage(%)	75.9 ± 19.1	73.7 ± 19.2	0.493
Pressure(mm Hg)	19.9 ± 1.0	39.6 ± 1.0	0.236
Wind speed(km/h)	6.7 ± 4.1	7.2 ± 4.6	0.625
Sunshine duration	654.7 ± 108.4	626.6 ± 95.5	0.050
UV index	3.3 ± 1.8	2.6 ± 1.1	0.044

*The values are presented as mean +/-SD.

As the mean age of migraine patients increased, the mean air temperature on the attack days decreased. It was observed that elderly individuals have migraine attacks in colder days. (r = –0.146 and p = 0.046) (Table 5)

**Table 5:** Relationship between weather parameters and age in migraine attacks.

	Age	
	r	p
Temperature	–0.146	0.046
Humidity percentage	0.117	0.110
Pressure	0.051	0.490
Wind speed	–0.005	0.949
Sunshine duration	–0.075	0.306
UV index	–0.113	0.122

## 4. Discussion

In the present study, we investigated the relationship between weather parameters and age in migraine attacks; as a result, it was found that, as the mean age increased, the mean air temperature of the days of the attacks decreased (r = –0.146 and p = 0.046). The frequency of attacks in cold weather was observed to increase in older ages. In a study by Hoffman et al., it was reported that migraine periods that started independently from any certain time of day were associated with low temperature and high relative humidity [7]. Although migraine attacks were found to be the highest in January and lowest in August, this finding was not found statistically significant. In the same study, the pathophysiological relation between air changes and the occurrence of migraine attacks was unclear, and it was indicated that some changes in specific air parameters may lead to an increase in the neuronal excitability of trigeminal neurons and thus initiate a migraine attack [7]. In another study, it was found that higher relative humidity was associated with higher odds of migraine headache onset in warm season [22]. 

In the study of Yılmaz et al., it was stated that the patients applied to the emergency department with migraine attacks mostly in November and December [5]. This is consistent with the present study. In the same study, although there was a positive correlation between the number of migraine patients and the average daily wind speed, it was stated that this finding was not statistically significant [5]. In the present study, it was shown that in migraine headaches, the mean wind speed on the attack days was significantly higher than the mean wind speed on the TTH attack days.

In the study of Tanik et al., in obese patients with tension-type headache, it was found that hot weather triggered headache more than cold weather. In the same study, in patients with migraine and tension-type headaches, no relationship was found between exposure to hot/cold weather as a trigger and the clinical features of headaches [23]. 

In present study, when the migraine and TTH attack days were compared in terms of temperature, wind speed, wind direction, humidity, pressure, UV index and sunshine duration, it was found that the mean wind speed in migraine headaches was significantly higher when compared to TTH. The mean UV index was found to be significantly higher in TTH attack days (p = 0.018 and p = 0.039, respectively). There was no significant difference in other parameters. There are no studies comparing migraine and TTH pains in terms of wind speed and UV index. Two studies by Spierings et al. and Wang et al. are similar to the present study in terms of headache types included in their studies. Migraine and TTH patients were included in both studies [12,13]. In the studies of Spierings, weather changes were found to be the triggering factors in 71% of migraine-type headaches and 35% of tension-type headaches [12]. In a study Wang et al. conducted with migraine and TTH patients, sunlight was in the third place in triggering factors with a rate of 32.7% in migraine group and with a rate of 20.9% in TTH group. In migraine patients, weather change was observed in the fourth place with a rate of 31.1%. Weather change was found to be the triggering factor in only 9.6% of TTH patients. In both migraine and TTH group, sunlight was indicated to be more triggering in females than in males, and wind in TTH was found to be more triggering in females than in males [13]. In the present study, no significant difference was found in the relationship between the mean wind velocity and sex in TTH attacks and the mean wind velocity was observed to be significantly higher in migraine-type headaches compared to TTH. The average UV index in TTH was found to be significantly higher in women and the mean sunshine duration was found to be significantly longer in women. 

In our study, no statistically significant relationship was found between air temperature and humidity and migraine and TTH attacks. In some studies, a relationship between temperature and humidity and headache was found, but in some others no relationship was found [5-14,15,18–19]. In an analysis of 7054 emergency department patients with a diagnosis of headache, it was found that high ambient temperature increased the risk of headache and this risk was higher in migraineurs [9]. In a retrospective study conducted by Yılmaz et al. with migraine patients presenting to the emergency department within a year, it was stated that migraine headaches increased with high temperature and low humidity, and the number of patients was not related to the phases of the moon [5]. 

In the present study, we could not find a significant relationship between wind direction and pain attacks of both groups. We thought this could have been due to geographical and climatic differences. Considering the fact that the winds blowing from the southeast (southeaster) and northwest (northwester), which are known to cause dry and hot air, are the prevailing winds in our region, our findings suggesting hot air is not directly related to headaches is supported. However, there are some studies showing that headaches are associated with various wind types [15,16,24]. In some studies, it was stated that the Chinook winds (fast and hot winds) in Alberta, Canada were associated with headache attacks [15,16]. In a study investigating the effects of Chinook winds in Calgary, Alberta, Cooke et al. found that the likelihood of migraine increased in the days before and during the rapid Chinook winds. Two subgroups were found to be sensitive in the days before the Chinooks and during the days of the Chinooks (only two patients were involved in both of these subgroups). Elderly patients were found to be more sensitive during the days of Chinooks than the days before Chinooks. In this study, 88% of the patients reported Chinook wind effects on their headaches with their own daily methods, but only 20% of them were proven accurate [16]. A study of Sharov winds in the Tel Aviv region showed no correlation between geomagnetic movements and headache frequency, but a linear correlation was found between these movements and headache severity [24].

In two different studies, the effect of changes in weather variables on headaches was investigated [6,8].In a study conducted in Taiwan with migraine patients without aura, Albert C. Yang et al. determined the headache incidence data associated with weather condition components through EMD (empirical mode decomposition) method which was used to detect timewise relations in the disorder, in the same study, weather conditions-time series was divided into pieces, and a significant relationship was found between headache incidence and weather condition parameters which could not be shown in analysis that was made with unprocessed weather condition data. In this study, the dates between 7th of August and 18th of October was determined as the hot period and the dates between 19th of October and 31st of December was determined as the cold period. Although many meteorological factors were found to have a strong correlation with the headache rate in the cold period, these relationships were observed to be weak or absent in the hot period. It was shown that increased temperature and sunshine duration following the cold air mass were responsible for a rate of 33.3% of the headache increase during the cold period. It was indicated that these findings support the notion that the changes in weather condition, especially during the cold period, cause headaches [6]. It was stated that cold weather masses in the cold period often caused a decrease in temperature and in sunshine duration, and an increase in barometric pressure and in relative humidity [25]. While the mechanism of the relationship between weather and headache was unknown, it was stated that as headache was associated with hemodynamic changes, cold air might have been affecting these changes [6]. In the study of Prince et al., it was indicated that most of the migraine patients who were weather-sensitive were also sensitive to temperature and humidity changes. In the same study, it was stated that some migraine patients were susceptible to multiple factors and that no difference was observed in the subgroup analyses that were conducted with regard to the triggering effect of sex, age, and weather conditions [8]. Based on these two studies, it is understood that the mechanisms that causes pain are also related to changes in weather parameters, so one can think that instant weather measurements are not sufficient during the investigation of the real effect of weather parameters on the characteristics of headaches. In clarifying the effect of weather parameters on the characteristics of headaches, the importance of questioning other factors that may be triggering before or during attacks should not be ignored. In the present study, we did not include patients’ perceptions in our analysis. In the studies that include subjective perceptions of the patients, the patients were found to be wrong in their perceptions. Prince et al. reported that most of the migraine patients perceived the weather as a triggering factor and that those in the analytical part of the study did not match the triggering weather conditions reported by the patients, and that most migraine patients, who were sensitive to the weather, were most sensitive to changes in temperature and humidity [8]. In the study of Iliopoulos et al. who questioned the triggering factors of headache, 46.55% of headache patients stated that they perceive air changes as a triggering factor. According to this study, hot or cold weather, wind and bright sunlight were regarded as significantly important triggers by migraine patients with aura. It was stated that the triggers should be decided together by the clinician and the patients [10]. In a study by Zebenholzer et al., in which the correlation between subjective air perception and meteorological data was calculated, only the effect of high pressure in increasing the risk of headache was found to be statistically significant. In the same study, no correlation was found between patients’ perception of weather and headache development. According to this study, the effect of weather on migraine is small, yet not negligible. In this study, when the relationship between migraine and weather was analysed, the importance of a study independent from subjective perceptions was emphasized [11]. In many studies, no statistically significant relationship was found between migraine headaches and weather parameters [17,18,19,20,21]. We believe that the differences among the subjective perceptions of the patients, the climate and weather differences in the regions of the studies, the current technical insufficiencies to evaluate the multilateral headache-weather relationship, the number of patients and inadequacies in the follow-up period lead to conflicting results obtained from the studies. 

We did not use the daily change rates of meteorological parameters and the measurements of meteorological parameters before pain, we rather made use of once-a-day weather condition data in our analysis and we did not question the other triggering factors other than the weather conditions; all these may prevent us from making a perfect analysis. Except for the comparison of the two groups in terms of descriptive characteristics, we treat each migraine attack or TTH patient as an incidence. Thus, we used 188 migraine attacks and 233 TTH attacks in our analyses. We could not analyse the weather parameters of the days of migraine or TTH attacks and of the days without attacks.

Since our study did not cover a full cyclic year, it did not include mid seasons. It would produce more reliable results if these inadequacies are fulfilled, the temporal coexistence of attacks and meteorological measurements are evaluated in more detail, multiple measurements are performed during the day and studies covering a longer period of time are conducted.

## 5. Conclusion

Based on the findings obtained in our study; we think that it may be useful to provide recommendations and warnings about weather conditions in the treatment and follow-up of migraine and TTH patients. We can say that this information can be guiding for the patients to regulate their daily living activities, and it should be noted that in case of unresponsiveness to treatment, considering the relationship between the weather and headache is of high importance during the review of the current treatment.

## Informed consent

Our research has started in compliance with the Helsinki Declaration resolutions, patient rights regulation, and codes of conduct after the approval given with the meeting resolution dated 17.06.2014 and numbered 2014/12 taken in Bülent Ecevit University Application and Research Hospital which was the local ethics committee.

All participants gave informed consent in the format required by the relevant authorities and / or boards.
